# Postarthroscopic Infection in the Knee following Medical or Dental Procedures

**DOI:** 10.1155/2013/974017

**Published:** 2013-09-26

**Authors:** Kat Swank, Jason L. Dragoo

**Affiliations:** ^1^Division of Sports Medicine, Department of Orthopaedic Surgery, Stanford University, Palo Alto, CA, USA; ^2^Department of Orthopaedic Surgery, Stanford University, Stanford University Medical Center Outpatient Center, 450 Broadway Street, Redwood City, CA 94063, USA

## Abstract

Knee infection is a relatively rare complication after arthroscopic knee surgery (Babcok et al., 2002), with reported incidences ranging from 0.1% to 3.4% (Babcok et al., 2002 and Bert et al., 2007). Although postoperative dental and surgical procedures were formerly considered a risk factor for infection in arthroplasty patients, the American Dental Association (ADA) and Academy of Orthopaedic Surgery (AAOS) no longer recommend prophylactic antibiotics for patients with prosthetic joints and do not make recommendations concerning patients who have recently undergone arthroscopic surgery (J. M. Bert and T. M. Bert, 2010). We report two patients who had an uncomplicated postoperative course until having a procedure and then developed a septic knee.

## 1. Methods

The records of all primary arthroscopic procedures carried out by the senior author (JD) between 2006 and 2012 were retrospectively analyzed. All postoperative infections were tallied, and patients were queried about the following known risk factors for postoperative infections: remote body-site infections, open wounds, rashes, postoperative medical/dental procedures, advanced age (50+ years of age), obesity, diabetes mellitus, altered immune response, systemic illness, lifestyle factors including nutritional status and tobacco use, and other factors including coexistent corticosteroid therapy, length of preoperative hospitalization, and colonization with microorganisms. Those patients who had a dental or medical procedure within 24 months of their arthroscopy were also asked to forward an extensive history of the dates of their medical or dental procedures directly from their physician or dentist. This study was approved by the Internal Review Board, and all subjects gave informed consent for their participation.

## 2. Results

Between January 2006 and December 2012, 2,174 patients had arthroscopic knee surgery performed by the senior author. Three patients developed a postoperative infection, indicating an incidence of 0.001%. Of those who developed an infection, 2 had risk factors identified on questionnaire, revealing that both patients had postoperative dental or medical procedures within 24 months of their arthroscopic procedure. The following is an account of their postoperative course.

## 3. Case Reports

### 3.1. Patient 1

The first patient is a 24-year-old female who complained of deep knee pain and pressure in her left knee as well as tenderness on the distal quadriceps tendon over a period of two years. She had been in physical therapy and taking anti-inflammatory medications for over six months and had not improved. Her past history was remarkable for significant sinus problems, including septal deviation and chronic right-sided sinusitis, for which she had surgery twenty-one days preoperatively.

The patient elected to proceed with arthroscopic surgery, which included anterior interval release, modified lateral release, synovectomy, and open quadriceps debridement. Postoperatively, the patient had no signs of discharge, erythema, and well-healing wounds with minimal pain at the two-week post-op visit. At that time, she had a check-up with her ENT for continued nasal congestion and pressure, but a head CT indicated her sinuses were clear, with no evidence of continuing infection or fullness.

At five weeks postoperatively, the patient returned to the office with no redness, tenderness to palpitation, and loss of, or pain with, range of motion (ROM), but minor posterior swelling. She had an MRI of the knee at that time, which showed no abnormality in the posterior knee, including edema, cyst, or abscess. A Doppler ultrasound was also negative for popliteal cyst, fluid collection, or deep venous thrombosis. Her physical examination was improving, and she continued with anti-inflammatory medications. 

She continued to improve until ten weeks postoperatively, when she underwent a series of dental procedures, including crown placement and root canal. The patient returned to the office three days after her root canal due to a marked, acute increase of pain and swelling in the knee. The physical exam illustrated no evidence of fluctuance or erythema; however, her knee was irritable with a painful ROM and a large effusion. An arthrocentesis was performed due a suspicion of infection. The aspirate contained a large number of polymorphonuclear cells, but no organisms were seen, and aerobic and anaerobic cultures showed no growth over 5 days. A cell count was not obtained due to a laboratory error. 

Due to her worsening exam and the high polymorphonuclear cell count in her synovial fluid, she was taken to the operating room for arthroscopic irrigation and debridement. During the arthroscopy, the knee appeared septic with fibrinous/purulent material present, but intraoperative cultures were negative. She was placed on antibiotics for six weeks. The patient continued to improve after the debridement and was markedly improved at one-year followup.

### 3.2. Patient 2

The second patient is a 43-year-old male with no chronic medical conditions who presented with left knee pain and effusion for approximately two years. MRI showed a medial and lateral meniscal tear and synovitis. He elected to undergo an arthroscopic partial medial and lateral meniscectomy and synovectomy. The patient was doing well at 2-week followup, with no fever, chills, or other signs of infection, and had a range of motion to 0–130 degrees and mild to moderate swelling. 

At 4 weeks and 35 weeks postoperatively, the patient underwent routine dental cleaning. However, at both the seven-week and nine-month followup, his effusion was markedly decreased from his preoperative state, and the patient noted a 60% improvement compared to before surgery. 

Thirteen months postoperatively the patient underwent nasal surgery consisting of an uncomplicated uvulopalatopharyngoplasty, tonsillectomy, and septal turbinoplasty. Less than three weeks later he had an acute increase in knee pain and swelling. Physical exam showed a large effusion and an exquisitely painful ROM arc. An arthrocentesis was performed due to suspicion for infection, which showed 7050/*μ*L WBCs and 88% neutrophils. No crystals, malignant cells, or intracellular microorganisms were seen, and there was no growth in the five-day aerobic and anaerobic cultures. 

He was taken to the operating room for left knee arthroscopy and irrigation and debridement due to clinically septic knee. Intraoperatively, the knee appeared septic with florid synovitis and fibrinous exudates. Intraoperative cultures remained negative. At one month followup postoperatively the patient was doing much better, with a scant effusion and minimal pain. 

## 4. Discussion

Complication rates for arthroscopy have been reported to be 0.1% to 3.4% [1, 2], with 0.01% to 0.48% of complications attributable to infection [1, 3, 4]. Our cohort infection rate of 0.001% is low and consistent with previous data. 

Surgery is generally indicated for postoperative infections in an attempt to decrease enzymatic injury to the articular cartilage [5]. In the majority of cases, patients experience onset of infection symptoms within 3 days of surgery, but patients can remain at risk for hematogenously induced infection for approximately 1 year postoperatively [4]. Patients with persistent pain and swelling following arthroscopy should have an arthrocentesis with synovial fluid analysis and culture regardless of presence or absence of erythema, fever, leukocytosis, or benign appearance of synovial fluid [4].

The “gold standard” for diagnosis of joint infection is culture of joint aspirate [4, 6]. However, because organisms are not always isolated from areas that ultimately prove to be infected, accurate diagnosis may necessitate the use of a combination of tests, including serologic tests measuring white blood-cell count, erythrocyte sedimentation rate (ESR), and C-reactive protein level [7, 8]. In the absence of underlying inflammatory joint disease, a synovial fluid leukocyte differential of greater than 65% neutrophils, or a leukocyte count of greater than 1.7 × 10^3^ white cells/*μ*L is considered diagnostic of periprosthetic knee infection [9, 10]. Researchers have suggested synovial fluid white blood cell count greater than 50,000 cells per *μ*L to be a useful discriminator of septic arthritis [11].

Risk factors for postoperative infection include remote body-site infections, recent surgical procedures, advanced age (50+ years of age), obesity, diabetes mellitus, altered immune response, lifestyle factors including nutritional status and tobacco use, and other factors including coexistent corticosteroid therapy, length of preoperative hospitalization, and colonization with microorganisms [5]. 

The American Dental Association (ADA) formerly recommended antibiotic prophylaxis for patients with orthopedic implants undergoing dental procedures. However, in 2012, the guidelines were revised, and the ADA and American Academy of Orthopaedic Surgery (AAOS) no longer recommend antibiotics for everyone with artificial joints [12, 13]. Similarly, the ADA has no recommendations for dental patients who have recently undergone arthroscopic surgery. The 2013 American Society of Health-System Pharmacists report does not recommended prophylactic agents for orthopaedic procedures for “clean operations involving hand, knee, or foot and not involving implantation of foreign materials [5]. 

However, the rate of bacteremia after dental and certain diagnostic or therapeutic procedures has been well documented, and the frequency and intensity of bacteremia associated with oral procedures are generally high [14]. Debelian et al. used phenotypic and genetic methods to trace microorganisms released to the blood stream during and after endodontic treatment and found that all root canals produced anaerobic bacteremia [15]. Depending on the extent of the procedure, organisms including *Propionibacterium acnes*, *Peptostreptococcus prevotii*, *Fusobacterium nucleatum*, *Prevotella intermedia*, *Saccharomyces cerevisiae*, *P. intermedia*, *Actinomyces israelii*, *Streptococcus intermedius*, and *Streptococcus sanguis* were isolated from the blood following root canals [15]. Another study of root canals identified 56 different microbial species and 21 different genera [16]. Although standard cultures are five days, Schäfer et al. found that prolonged microbiological culture of synovial fluid over a 2-week period yielded signs of periprosthetic infection in a significant proportion of patients whose periprosthetic infection would have otherwise remained unidentified [17]. Additionally, late hematogenous infection caused by flora indigenous to the body, and temporally related to these invasive procedures, has been documented [18]. An investigation of sources of periprosthetic infection done by Bartzokas et al. found that the strain of *S. sanguis* isolated from the prosthesis was indistinguishable from the strain isolated from the mouth [19]. This may be due to increased blood flow to the synovial membrane following surgery [20]. It is possible that these pathogens could be hematogenously seeded into the joint following dental procedures causing infection among patients who have recently undergone knee arthroscopy. Similarly, organisms including *Streptococcus pneumoniae*, *Haemophilus influenzae*, *Moraxella catarrhalis*, *Streptococcus pyogenes*, or *Staphylococcus aureus* have been isolated from nasal cultures in patients with rhinosinusitis [21]. It is possible that bacteria liberated from the sinus cavities during surgical treatment could likewise be seeded into the joint hematogenously. 

In a review of the literature, Babcock et al. found that the most commonly reported causative organisms of postarthroscopic joint infection are *Staphylococcus* species [1], while Armstrong et al. reported knee joint infections are most often due to *Staphylococcus aureus*, coagulase-negative *staphylococci*, and *Enterobacter cloacae *[4]. 

## 5. Conclusion

Currently, the American Society of Health-System Pharmacists, American Dental Association, and American Academy of Orthopaedic Surgery do not recommend prophylactic antibiotics for patients who have recently undergone knee arthroscopy or arthrotomy. In the cases discussed here, neither patient had postoperative symptoms concerning infection following arthroscopy. However, patient 1 developed infection of the joint shortly after undergoing a dental procedure, despite being placed on antibiotics by her dental surgeon. Patient 2 developed infection three weeks after undergoing nasal surgery. Although these are isolated incidents, they suggest that future studies investigating incidences of postoperative joint infection following dental and surgical procedures are warranted. Additionally, 5-day cultures remained negative in these cases, so additional culture time up to 21 days may be warranted.
